# Technical Education in London

**Published:** 1897-07-10

**Authors:** 


### Modern Sociology.

### TECHNICAL EDUCATION IN LONDON.

The City and Guilds of London Institute for the Advancement of Technical Education was founded in 1878. After close upon a quarter of a century of work, it may be held to have stood the test of time; and yet little more than a year ago the most serious charges of waste, extravagance, and futility were brought against it in an anonymous pamphlet. The strength of an anonymous pamphlet lies in its anonymity. When you know who brings an accusation, you can measure its justice; but the accuser who does not reveal himself is as formidable as the Vehmgericht of the Middle Ages. He can strike, but it is difficult to return the blow. The council of the institute did the best thing they could when they referred the matters dealt with in the pamphlet to a committee of investigation. That committee has now presented its report, and in so doing has given a succinct account of the working of the institute.

The institute commenced its work in temporary premises in some rooms in the basement of Cowper Street Schools. It was from the first intended to house the institute in some suitable college building, with proper class-rooms and laboratories, but it was not thought necessary to postpone the commencement of its work till a home should be provided for it. It was hoped that the Corporation of the City might grant a site for the college. This hope was not realised, and, as the price of land within the City was prohibitive, the members of the institute were glad, two years after, to accept a site from the commissioners of the exhibition of 1851, a plot of ground in Exhibition Road, which was leased at a peppercorn rent on the condition that the building erected should cost not less than £50,000, and that £5,000 a year should be spent on its maintenance. This was a costlier building than the institute had originally intended—£30,000 to £35,000 was the amount first suggested for the purpose—but the practical gift of the site made it not injudicious to accept the conditions imposed. What was spent on the building was saved on the site. Moreover, the site was in itself a very desirable one, as it is near South Kensington Museum, where there is a magnificent collection of models and examples of machinery, and also of teaching apparatus. It is also near Hyde Park, where students in civil engineering get practice in surveying during the summer, and it is near the Royal College of Science. These are all advantages so great as to counterbalance the facts, dwelt on by the anonymous fault-finder, that the building has cost more than was originally suggested, and that, although a City foundation, it is not in the City.

As a matter of fact, it is rather foolish than wise to put educational establishments in the modern City of London. Almost no one lives in the City now; it is a vast business office, and the atmosphere is not conducive to study. This is so generally felt that the great City schools, which have dwelt within its bounds for centuries, are now being removed to the suburbs, and it would have been a mere sacrifice to a name to have placed the Institute for Technical Education there. Kensington is for nine-tenths of the students as easy of access as the City, and in every other way all the advantages lie with it.

The building as it now stands cost £79,200, and its equipment £22,600; in all £101,800. Large as this sum may seem, it is less than that of similar institutions. The nearest to it is University College, Liverpool, which cost £128,750; the farthest away, Cornell University, in the state of New York, which cost over half a million sterling. At Cornell a large amount of money has been spent on models of machines, &c. The London Institute is saved this by having South Kensington so near.

The Institute is not a self-supporting institution; that must be understood. It was never expected to be so, for the intention of it, as of all our great colleges, was to enable young people to obtain an education which, in most cases, their own or their parents' resources could not afford. Free education for the masses is a new idea, but assisted education is a very old one; and assisted education, because it leaves something for the pupils to do, because it often demands some sacrifice, is more generally appreciated than free. It was estimated when the City of London Institute was established, that the cost of maintaining 200 students at it would be £10,000 a year, even though fees amounted to £5,000 a year. That is, it was expected that the students would pay only a third of the cost of their education. As a matter of fact, in 1895, the last year of which we have complete statistics, they paid nearly half—£4,993, out of £11,682. As many of the expenses are permanent, an increase in the number of students would tend still further to decrease the cost *per capita* to the institute. Even as it stands, however, the net cost per student (£31 a year) is by no means excessive. It is £19 less than the original estimate, and yet the fees charged are rather less than in similar colleges elsewhere.

The professors at the Central Technical College receive a fixed salary of £1,000. In many places it is customary to give the professors a smaller salary, and a share in the fees of students. The danger of this latter course is that the professor is tempted to lower the standard of acquirement demanded from students on entering the college, in order to have larger classes and more fees. Man is but mortal, and to get his best and most unselfish work, it is wise to remove the temptation to selfishness. It has been found, too, in all colleges that many students take only those classes which have a direct bearing on their future profession. This often causes great differences between the salaries of equally able and earnest teachers. A certain equality of income is desirable and fitting between men who are of the same rank and status, and all recent university reform has gone on the lines of fixing the salary of professors. For a man such as the institute desires for a professor in its college an income of £1,000 is by no means too much. On the other hand, the salaries of the demonstrators, who assist the professors, is purposely kept low, £250, in order that young men of promise may not be tempted to spend their lives in a subordinate position, but may go elsewhere to better themselves.

One of the complaints of the nameless foe is that the students of the college do not, on completing their course of study, at once get remunerative employment, but have often to undergo an apprenticeship to their chosen profession. He evidently confounds technical education with manual training. It is after the apprenticeship that the technical education tells. As the committee say, judgment should not be passed on the work of the college by the positions which students obtain immediately on leaving, but by those which they obtain within the next ten years.

